# Spider Web-Inspired Graphene Skeleton-Based High Thermal Conductivity Phase Change Nanocomposites for Battery Thermal Management

**DOI:** 10.1007/s40820-021-00702-7

**Published:** 2021-08-18

**Authors:** Ying Lin, Qi Kang, Han Wei, Hua Bao, Pingkai Jiang, Yiu-Wing Mai, Xingyi Huang

**Affiliations:** 1 Shanghai Key Lab of Electrical Insulation and Thermal Ageing, The State Key Laboratory of Metal Matrix Composites, Department of Polymer Science and Engineering, Shanghai Jiao Tong University, Shanghai, 200240 People’s Republic of China; 2 University of Michigan-Shanghai Jiao Tong University Joint Institute, Shanghai Jiao Tong University, Shanghai, 200240 People’s Republic of China; 3 Centre for Advanced Materials Technology (CAMT), School of Aerospace, Mechanical and Mechatronic Engineering J07, The University of Sydney, Sydney, NSW 2006 Australia

**Keywords:** Thermal conductivity, Radial freeze-casting, Phase change materials, 3D graphene aerogel, Thermal management

## Abstract

Phase change materials (PCMs) can be used for efficient thermal energy harvesting, which has great potential for cost-effective thermal management and energy storage. However, the low intrinsic thermal conductivity of polymeric PCMs is a bottleneck for fast and efficient heat harvesting. Simultaneously, it is also a challenge to achieve a high thermal conductivity for phase change nanocomposites at low filler loading. Although constructing a three-dimensional (3D) thermally conductive network within PCMs can address these problems, the anisotropy of the 3D framework usually leads to poor thermal conductivity in the direction perpendicular to the alignment of fillers. Inspired by the interlaced structure of spider webs in nature, this study reports a new strategy for fabricating highly thermally conductive phase change composites (sw-GS/PW) with a 3D spider web (sw)-like structured graphene skeleton (GS) by hydrothermal reaction, radial freeze-casting and vacuum impregnation in paraffin wax (PW). The results show that the sw-GS hardly affected the phase transformation behavior of PW at low loading. Especially, sw-GS/PW exhibits both high cross-plane and in-plane thermal conductivity enhancements of ~ 1260% and ~ 840%, respectively, at an ultra-low filler loading of 2.25 vol.%. The thermal infrared results also demonstrate that sw-GS/PW possessed promising applications in battery thermal management.

**Supplementary Information:**

The online version contains supplementary material available at 10.1007/s40820-021-00702-7.

## Highlights

- A three-dimensional spider web-inspired structured graphene skeleton is constructed.
- The spider web-like skeleton endows paraffin wax with a significantly high longitudinal and transverse thermal conductivity enhancement of ~1260% and ~840%, respectively, at 2.25 vol% skeleton.
- The resultant composites exhibit outstanding performance on the thermal management of Li-ion batteries.

**Supplementary Information:**

The online version contains supplementary material available at 10.1007/s40820-021-00702-7.

## Graphical abstract

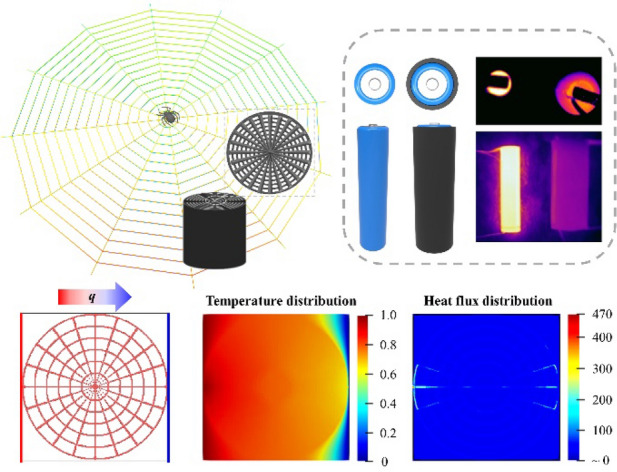

## Introduction

Efficient heat dissipation becomes a crucial problem with the development of miniaturization and high integration of electronic devices [1–5]. Overheating of these devices during operation would reduce the lifespan and reliability and even cause fire risks [6–8]. In the last few decades, phase change materials (PCMs) have been widely applied in many fields of building energy conservation, solar-energy storage, thermal management of electrical and electronic devices, etc., owing to their characteristic of absorbing and releasing thermal energy during phase transitions [9–12]. However, similar to most other polymeric materials, pristine PCMs show a low intrinsic thermal conductivity, which significantly influences the performance efficiency of PCMs in application systems. Additionally, problems of leakage at relative high temperatures can also be an important issue for PCMs [13–15].

Undoubtedly, thermal conductive property is one of the most considered performance factors in thermal management. The traditional methods for enhancing thermal conductivity of PCMs are to add highly thermally conductive fillers to the matrix, such as carbon materials, ceramics and metal nanoparticles [16–19]. Carbon materials (e.g., graphite nanoplatelets, graphene, graphene oxide (GO) and expanded graphite (EG)) have received growing interest due to their super-high thermal conductivity and easy availability [20–25]. In general, two-dimensional (2D) nanofillers tend to display large differences between lateral size and thickness [26–29]. When these individual nanofillers are dispersed in a PCM, the thermal conductivity enhancement of the composite can be less obvious because of the large thermal resistance arising from the random geometric contacts below the percolation network [30, 31]. Usually, dispersed fillers can give extremely unstable thermal conductivities of phase change composites (PCCs) during their solid–liquid phase transitions. To solve these problems, 2D nanofillers are constructed as continuously aligned structures with different processing methods of three-dimensional (3D) printing, mechanical compression, ice template assembly or electric/magnetic field-assisted orientation [16, 20, 32]. It is noted that 3D porous carbon materials can serve as continuous thermally conductive frameworks in PCCs even at relatively low filler loading, hence providing more efficient phonon transfer pathways. Simultaneously, these frameworks have large specific surface areas, which can absorb molten PCMs by means of capillary and surface tension forces to reduce the leakage [12, 33]. Min et al. reported PCCs containing anisotropic graphene aerogel as the framework demonstrated excellent transversal and longitudinal thermal conductivities with obvious simultaneous anisotropy [10]. In another study, a porous hybrid graphene aerogel endowed PCCs with not only high thermal conductivity of 5.92 W m^−1^ K^−1^ but also good shape stability, thus avoiding the leakage of molten PCM [33]. Sheng et al. prepared a vertically aligned carbon fiber scaffold by using a facile method and the obtained PCCs displayed an enhanced thermal conductivity of 0.77 W m^−1^ K^−1^ in the axial direction at a skeleton loading of 8.8 wt%. In addition, these PCCs exhibited good shape stability against leakage at temperatures above the melting point of the PCMs [15].

In fact, highly thermally conductive graphene is frequently used to enhance the thermal conductivity of PMCs [9, 34]. As a derivative of graphene, graphene oxide (GO) can form liquid crystals (LCs), which can be precisely organized into 3D ordered microstructures by self-assembly of their building blocks [35–37]. Although highly aligned architectures are conducive to the thermal conductivity enhancement of polymer composites, their anisotropy easily results in severely weak thermal conductive performance in the direction perpendicular to the orientation of the filler network [38].

Herein, inspired by the spider web’s (sw) interlaced structure, a 3D graphene skeleton (GS) was so constructed by using hydrothermal reaction and radial freeze-casting. The graphene aerogel served as the heat conductive framework for paraffin wax (PW). This 3D interlaced architecture reduces phonon scattering and effectively improves the thermal conductivity of the paraffin composites, sw-GS/PW, especially in the longitudinal (i.e., cross-plane) direction. The superiority of thermal conductivity in transverse direction is further confirmed by simulation. Also, the sw-GS can effectively restrain the melt leakage of PCCs at temperatures above the melt temperature. When these PCCs are wrapped around a battery, significant temperature drops can be achieved during charge and discharge. These encouraging results indicate great potential for thermal management of electronic and electrical devices.

## Experimental Section

### Materials and Methods

#### Materials

Graphene oxide (GO, slurry) was prepared using the modified Hummers method; paraffin wax (PW, melting temperature ~ 47 °C, C_n_H_2n+2_, *n* = 18 ~ 30) was purchased from Sinopharm Chemical Reagent Co., Ltd (China); and deionized water (DI water) was made in the laboratory.

#### Preparation of Spider Web-inspired Graphene Skeleton (sw-GS)

GO slurry was first treated by ultrasonic dispersion for 0.5 h, then mixed with certain amounts of potassium hydroxide aqueous solution by magnetic stirring for 0.5 h to obtain a well-dispersed suspension with ~ 40 mg mL^−1^ GO and 0.135 M KOH. The alkaline suspension was transferred to a hydrothermal reactor at 180 °C for 3 h to form a hydrogel. The produced hydrogel was dialyzed to neutral in deionized water, followed by freeze-casting with a homemade metal mold in liquid nitrogen, and then freeze-dried for 72 h. The resultant graphene skeleton was prepared by annealing the freeze-dried aerogel at 1500 °C for 1 h in an argon protection environment.

#### Preparation of Spider web-Inspired 3D Graphene/Paraffin (sw-GS/PW) PCCs

A container with the right amount of PW was placed in a 65 °C oven for 0.5 h, the graphene skeleton was immersed completely in the molten wax. By using vacuum impregnation, the aerogel was fully impregnated with the melt. The PCCs could be obtained by cooling the filled skeletons slowly to room temperature. The resultant PCCs are marked as sw-GS/PW-x, where x represents the volume fraction (%) of sw-GS in the composites. For example, sw-GS/PW-1.0 contains 1.0 vol% loading of sw-GS.

#### Preparation of PCC Wrapped Battery

A cylindrical hole with the same diameter of the battery was cut in the middle of a cylindrical sw-GS/PW-2.25 sample so as to house the battery. To increase the contact between the battery and PCC, the PCC wrap was heated to soften and then compressed with an annular mold. After cooling to room temperature, a PCC wrapped battery was obtained.

### Characterization

Arrangements of GO sheets in aqueous suspensions with different pH values were characterized by a polarized light optical microscope (POM, Leica DM LP, Germany) in transmission mode. In this case, a droplet of GO suspension was deposited on a glass slide between a pair of crossed polarizers for observing and collecting the POM images. The X-ray diffraction (XRD) patterns were recorded using an X-ray diffractometer (D8 Advance, Bruker, Germany). The microstructures of the graphene aerogels and PPCs were characterized with a field emission scanning electron microscope (FE-SEM, Nova Nano-SEM 450, FEI, USA). The cross-sectional profile for SEM examination was obtained by cryo-cutting the samples in liquid nitrogen. The phase transformation results of PW and PCCs were obtained using differential scanning calorimetry (DSC, Netzsch 200 F3, Germany) under a nitrogen atmosphere at a heating/cooling rate of 5 °C min^−1^. The thermal diffusivity (*λ*) (mm^2^/s) was measured with a light flash apparatus (LFA467 HyperFlash, Netzsch), and the thermal conductivity (*κ*) W m^−1^ K^−1^ was calculated by: *κ* = *ρ* × *C*_*p*_ × *λ*, where *ρ* and *C*_*p*_ represent the density (g cm^−3^) and specific heat (J g^−1^ K^−1^) of the sample, respectively. The diameter and thickness of the thermally conductive samples were 12.7 and ~ 2 mm, respectively. A sketch of heat conduction direction in testing and orientation of the graphene skeleton in PCCs is shown in Fig. S1. The change of surface temperature of battery during charging and discharging was recorded by an infrared thermal camera (FOTRIC 220).

## Results and Discussion

### Fabrication and Microstructures of 3D Spider web-Inspired Graphene Skeleton (sw-GS) and Its Phase Change Paraffin Wax (PW) Composites (sw-GS/PW)

As a derivative of graphene, GO acts as an important precursor in chemically modified graphene. It can be produced inexpensively by oxidation and exfoliation of natural graphite flakes. The GO nano-sheets in this study were prepared by the modified Hummers method [39]. As reported, aqueous GO can form liquid crystals (LCs), which is very important for achieving ordered microstructures of graphene-based materials [40, 41]. Since the liquid crystal characteristic of the GO depends on its size, solid content of the aqueous suspension and viscosity of the solution, etc. [42, 43], this means that highly ordered microstructures can be constructed under certain conditions. To minimize the impact of other factors, potassium hydroxide (KOH) was added to the GO suspension to induce the formation of the GO LCs. Figure S2 shows the POM images of 0.5 wt% GO suspension on neutral and alkaline conditions. Compared to the neutral system, the GO suspension containing 0.135 M KOH exhibited highly ordered hierarchical microstructures, displaying prominent liquid crystal behavior. It has been reported that the liquid crystal characteristic is often reflected in subsequent materials [36].

**Fig. 1:**
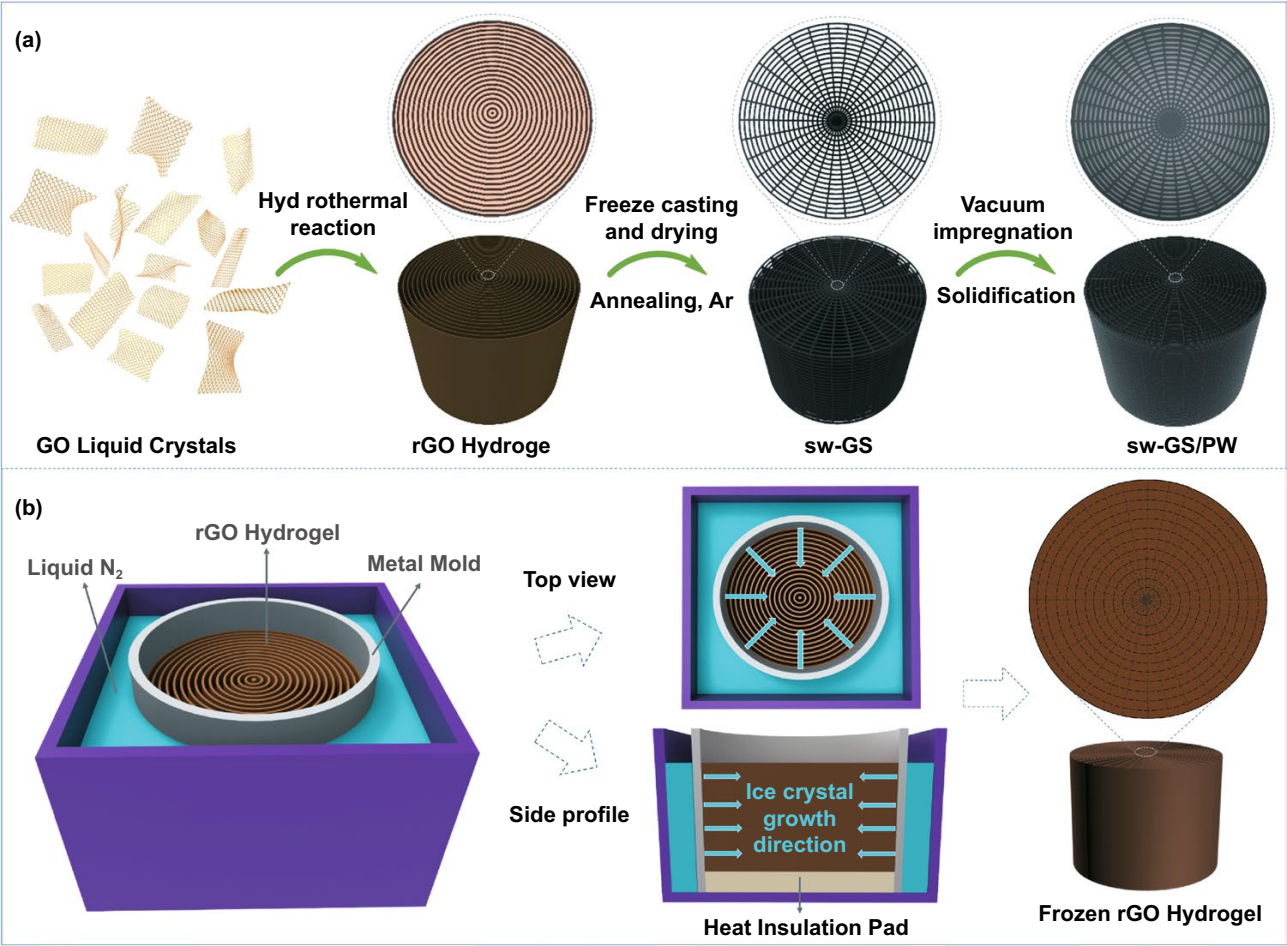
**a** Schematic illustration for preparation of polymer composite, sw-GS/PW. **b** Schematic diagram of radial freeze-casting for rGO hydrogel

**Fig. 2:**
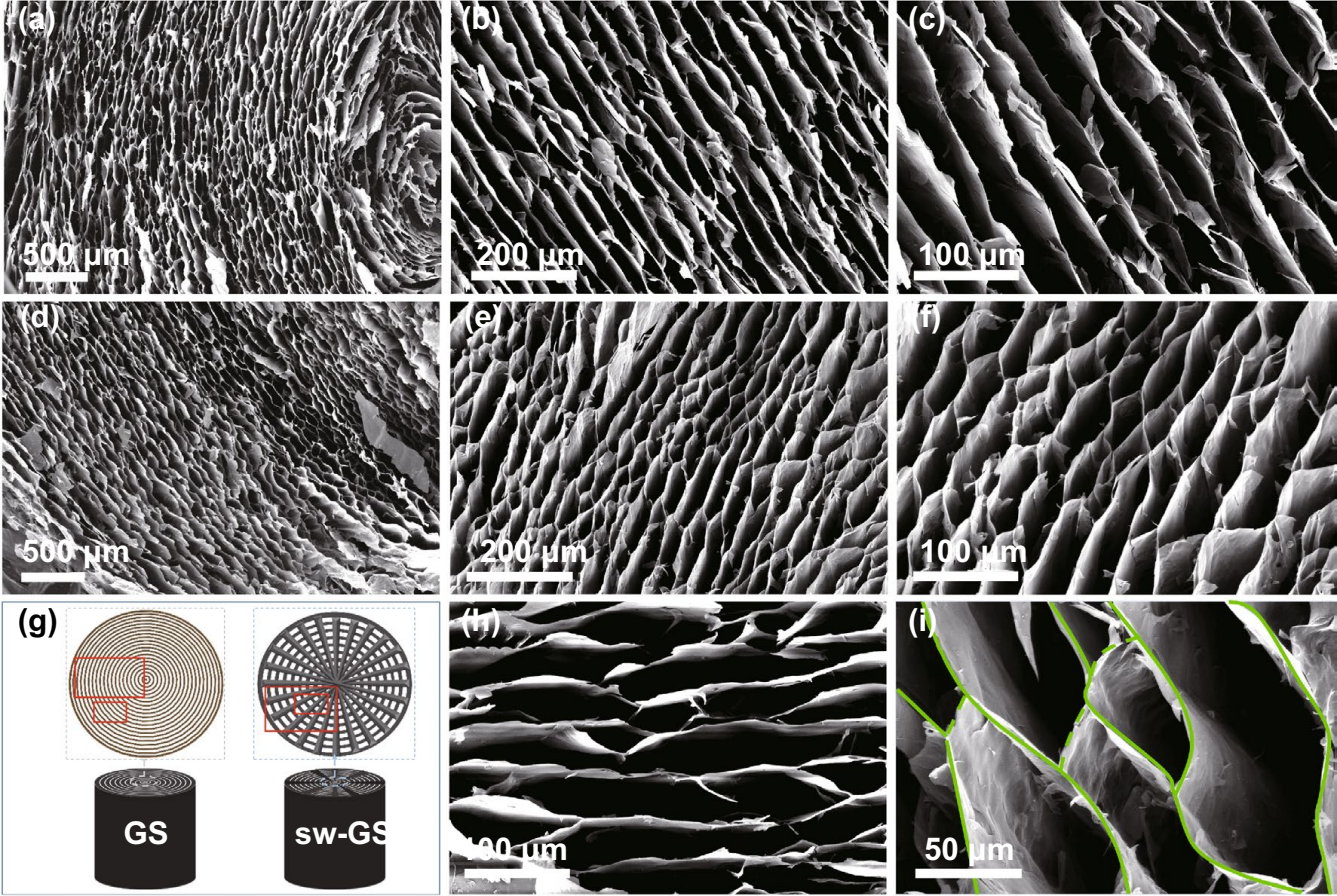
SEM images of rGO aerogels prepared by **a**–**c**, **h** unidirectional freeze-casting and **d**–**f**, **i** radial freeze-casting at different magnification. **g** Schematic illustrations of morphologies for GS and sw-GS, respectively. The SEM images displayed in **a**–**f** and **h–i** correspond to the regions marked with rectangular boxes in GS and sw-GS. The interlaced structure of sw-GS in **i** is outlined in green lines. (Color figure online)

As shown in Fig. 1a, 3D graphene hydrogel was obtained by the self-assembly of GO suspension containing KOH. During hydrothermal reaction, the alkaline GO LCs evolved to an orderly organized laminar texture with highly aligned bands. Since the hydrothermal reaction temperature was ~ 180 °C, GO was converted to reduced GO (rGO) during this process (Fig. S3). To maintain the original microstructures of the hydrogel, unidirectional freeze-drying was employed to fabricate the aerogel from the hydrogel (Fig. S4). The direction of freeze-casting is shown in Fig. S4b. Since the ice crystals grew along the direction of the laminar wall in the graphene hydrogel during freeze-casting, the original hydrogel structure was unaffected by freeze-drying and the interlaminar structure was preserved in this process. SEM images of rGO aerogel prepared using unidirectional freeze-drying in Fig. 2a–c revealed the original micromorphology of the hydrogel made from GO LCs. (The SEM images displayed in Fig. 2a–c correspond to the regions identified by the rectangular boxes in GS in Fig. 2g left.) As shown in Fig. 2a, the onion-like microstructure with rGO sheets are well organized as concentric rings in the aerogel cross section. But large gaps between neighboring rGO walls can be observed, and these walls hardly bridge each other (Fig. 2b, c, h). This long-range ordered microstructure seems to have inherited from that of the GO LCs. Even though a highly aligned 3D heat conductive framework increases the thermal conductivity of polymer composites, the concentric graphene skeleton clearly reveals disconnected microstructures throughout the cross section which, in turn, would discourage phonon transmission in the transverse (in-plane) direction of the composites.

Inspired by the interlaced structures of spider webs, it was thought useful to improve the connections between adjacent rGO lamellae within the cross-section of the concentric graphene aerogel. This could enhance the in-plane heat conduction of composites containing such 3D network. Figure 1b (left) shows a homemade mold for radial freeze-casting. The cylindrical mold was made of metal, but its bottom was filled with heat-insulated polymeric materials. When freeze-casting was conducted, only the circumference of the mold was in direct contact with the cold source (liquid nitrogen), thus yielding a radial temperature gradient. Hence, the ice crystals grew radially from the periphery to the center in the cylindrical hydrogel. This would promote deformation of rGO walls and adhesion between adjacent walls of the hydrogel. In this situation, the microstructures of the hydrogel were converted from the unconnected concentric rings to interlaced networks after freeze-drying (Fig. 1b). Microstructures of corresponding parts of the resultant aerogel are shown in Fig. 2d–f, i. The SEM images displayed in Fig. 2d–f correspond to the regions marked with rectangular boxes in sw-GS in Fig. 2g right.

Clearly, the onion-like morphology suffered radial extrusions of the ice crystals so that the freeze-dried hydrogel (i.e., aerogel) exhibited spider web-like structures in the cross section. The aerogel displayed a long-range ordered main structure and its neighboring rGO walls were bridged, forming an interlaced 3D network. As shown in Fig. 2i, the specific connections between rGO walls were outlined with green lines. These results confirmed the freeze-casting direction controlled the microstructures of the aerogel (as evidenced for GS and sw-GS in Fig. S5a, b). Thus, the freezing direction was critical to forming the spider web-like structure. In addition, the freezing temperature also has a significant influence on the growth rate of the ice crystals, affecting the pore size of the sw-GS sample [44]. It was found that the lower the freezing temperature, the smaller the pore size. Therefore, liquid nitrogen was used as the cold source for freeze-casting in this work. The biomimetic structures obtained have high connection densities, which enable their broad applications for fast efficient heat dissipation.

Phase change composites (e.g., sw-GS/PW) were fabricated from the 3D spider web-like graphene skeleton (sw-GS) and paraffin wax (PW) by vacuum impregnation as fully revealed in Fig. 1a. The graphene skeleton should be annealed at high temperature in an argon atmosphere to remove oxygen-containing groups to reduce phonon scattering. The final aerogel had a spider web-like microstructure (Fig. S5c). As for sw-GS/PW, its skeleton (sw-GS) retained the interlaced microstructure after impregnation by PW; however, the micropores of the graphene skeleton became rounded (Fig. S5d) plausibly caused by the infiltration and solidification of the molten paraffin.

### Thermal Performances of sw-GS/PW

**Fig. 3:**
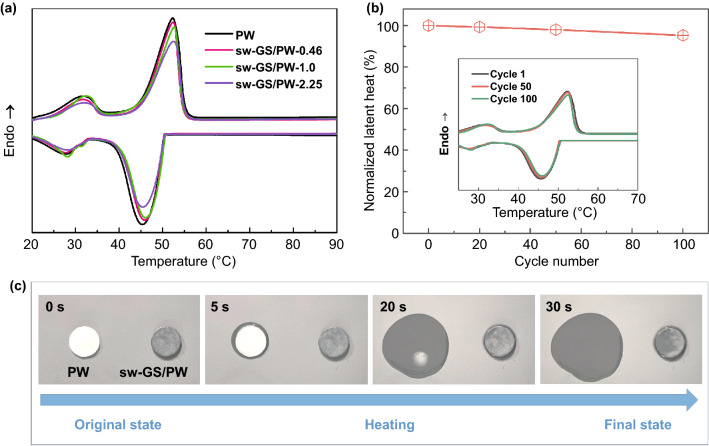
**a** DSC curves of PW and sw-GS/PW composites. **b** Normalized latent heat of fusion for sw-GS/PW-2.25 after thermal cycling (inset shows the DSC curves of sw-GS/PW-2.25 up to 100 thermal cycles). **c** Optical photographs of PW and sw-GS/PW-2.25 on a 65 °C hot plate

To examine the effect of graphene skeleton on the thermal properties of the phase change composites, three samples containing different sw-GS loading of 0.46, 1.0, and 2.25 vol% and designated sw-GS/ PW-0.46, sw-GS/PW-1.0, and sw-GS/PW-2.25, respectively, were fabricated. Their thermal properties were characterized by differential scanning calorimetry (DSC). Figure 3a displays the DSC curves of these three PCCs, all showing two peaks at ~ 30 and ~ 50 °C which resemble the characteristic peaks of pure PW and correspond to the solid–solid phase transition and solid–liquid phase change [45]. The melting temperature (*T*_*m*_) and latent heat of fusion (Δ*H*_*m*_) of pure PW were 52.2 °C and 191.4 J g^−1^, and the solidification temperature (*T*_*s*_) and solidification latent heat (Δ*H*_*s*_) were 45.3 °C and 176.1 J g^−1^, respectively (Table 1). Δ*H*_*m*_ and Δ*H*_*s*_ of sw-GS/PW decreased slightly with increasing sw-GS loading, but remained at a relatively high level. For example, Δ*H*_*m*_ and Δ*H*_*s*_ of sw-GS/PW-2.25 retained over 90% of those of pure PW. Moreover, *T*_*m*_ and *T*_*s*_ of sw-GS/PW did not change much compared to those of pure PW, suggesting low sw-GS loading had little effect on the phase transition behaviors of these PCCs. Figure 3b shows the DSC curves of sw-GS/PW-2.25 after melting–solidification cycles. It reveals that even after 50 and 100 cycles, the DSC curves of sw-GS/PW-2.25 remained stable and almost coincided with the original curve (1 cycle). Also, as demonstrated in Fig. 3b, the latent heat of fusion after 100 cycles remains at 93.5% of its original value, which shows an excellent latent heat cyclic stability of sw-GS/PW-2.25.

**Table 1:** DSC heating and cooling characteristics of paraffin and composites

Sample/composition	*T*_*m*_ (°C)	Δ*H*_*m*_ (J g^−1^)	*T*_*s*_ (°C)	Δ*H*_*s*_ (J g^−1^)
PW	52.2	191.4	45.3	176.1
sw-GS/PW-0.46	52.4	187.2	45.9	171.3
sw-GS/PW-1.0	52.7	184.6	46.0	169.9
sw-GS/PW-2.25	53.5	172.5	45.4	158.9

Thermal stability is critical for the PCCs to retain good performance during their service operations. Generally, a good shape stability can effectively prevent the leakage of molten PCMs from the PCCs [15, 46]. To evaluate this property, sw-GS/PW-2.25 and pure paraffin wax were put on a 65 °C heating stage. It can be observed from Fig. 3c, paraffin wax lost its shape quickly during the heating process and spread out on the heating stage. However, sw-GS/PW-2.25 retained its shape at 65 °C and there was no obvious leakage of molten paraffin, indicating that sw-GS effectively improved the shape stability of the PCCs. The excellent stability of sw-GS/PW-2.25 could come from the supporting microstructure of the 3D graphene skeleton owing to the capillary forces of its micropores which absorbed the molten PCMs during heating [10, 45].

### Thermal Conductivity of sw-GS/PW

**Fig. 4:**
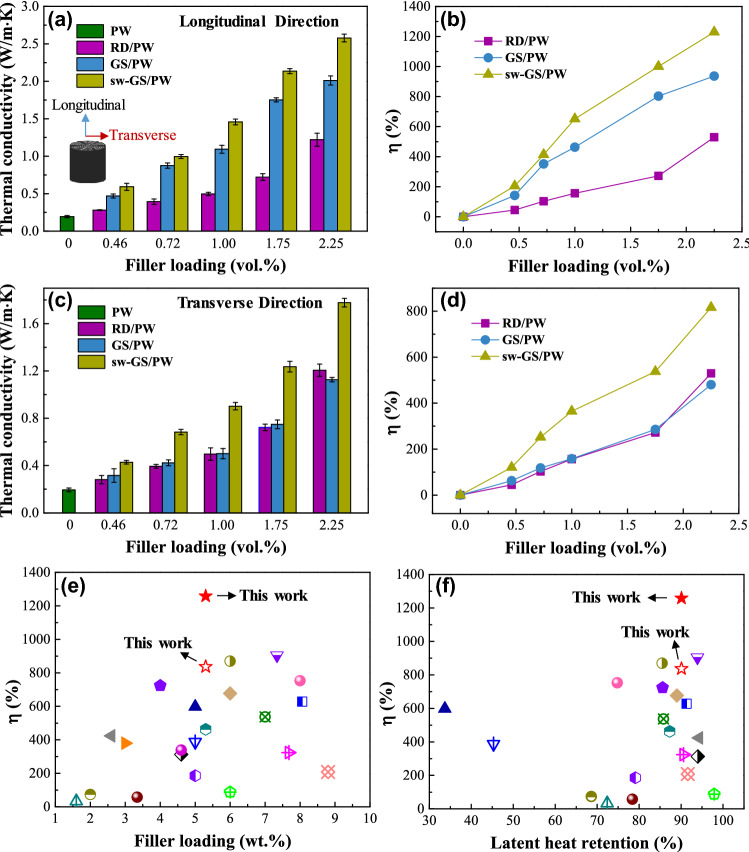
**a** Thermal conductivity and **b** enhancement in longitudinal (cross-plane) direction of PW composites; inset in (a) shows the directions of thermal conductivity for PW composites. **c** Thermal conductivity and **d** enhancement in transverse (in-plane) direction of PW composites. **e** Comparison of thermal conductivity enhancement (*η*) of sw-GS/PW-2.25 with those of other reported PCCs having low filler loading (< 10 wt.%) [15, 17, 19, 34, 47–62]. **f** Comparisons of thermal conductivity enhancement (*η*) and latent heat retention of sw-GS/PW-2.25 with those of other reported PCCs with relatively low filler loading [15, 17, 19, 34, 47, 48, 50–62]

High-performance PCCs need high thermal conductivity to accelerate the phase transitions of PCMs for efficient energy storage and release. Thermal conductivity was calculated from thermal diffusivity (see Fig. S6) measured by the light flash method at room temperature. For comparison, three different types of PCCs (RD/PW, GS/PW and sw-GS/PW) were prepared. RD/PW was fabricated by blending crushed sw-GS (powder) with paraffin wax (PW) matrix and solidification, the fillers were uniformly dispersed in PW. In addition, GS/PW and sw-GS/PW, the long-range ordered concentric graphene aerogel and the spider web-inspired graphene skeleton, were also used. Figure 4 shows the thermal conductivities and enhancement of these PCCs. The thermal conductivity enhancement factor (*η*) is calculated from Eq. (1):

1 $$ \eta = \frac{{\kappa_{{{\text{PCC}}}} - \kappa_{{{\text{PW}}}} }}{{\kappa_{{{\text{PW}}}} }} \times 100\% $$

where κ_PCC_ and κ_PW_ are the thermal conductivity of PCCs and PW, respectively; η is either cross-plane (longitudinal) or in-plane (transverse) thermal conductivity enhancement factor defined by the relative direction to the skeleton orientation (see inset in Fig. 4a). The thermal conductivity of pure PW was 0.19 W m^−1^ K^−1^. This low intrinsic thermal conductivity would limit the heat conduction in the phase change material and hence low efficiency in energy storage and release. By contrast, the PCCs showed higher cross-plane thermal conductivities with increasing filler loading. Specifically, with 2.25 vol% filler, the cross-plane thermal conductivities of RD/PW, GS/PW, and sw-GS/PW exhibited 1.21, 2.01, and 2.58 W m^−1^ K^−1^, respectively, yielding approximately 537, 958, and 1258% enhancement compared to PW (see Fig. 4a, b). Since the spider web-like graphene skeleton, sw-GS, had more connections in the in-plane direction than the 3D concentric ring skeleton, GS, this effect on the in-plane thermal conductivity of PCCs was also investigated. Figure 4c, d depicts the in-plane thermal conductivity and enhancement of the PW composites with filler loading. Since RD/PW has randomly dispersed fillers, it displayed isotropic thermal conductivity. However, 3D PCCs of GS/PW and sw-GS/PW exhibited anisotropic thermal conductivity directly reflecting the anisotropy of the filler skeletons in the matrix. Similar to the cross-plane thermal conductivities, the in-plane thermal conductivities of the PCCs were also enhanced with increasing filler loading. At the same filler loading, sw-GS/PW had the highest in-plane thermal conductivity and substantially outperformed RD/PW and GS/PW due to the interlaced structure of the sw-GS skeleton having higher density of in-plane heat conduction pathways than GS and RD. It might be fortuitous that RD/PW and GS/PW composites possessed similar in-plane thermal conductivities at the same filler loading.

Moreover, the low filler (i.e., graphene skeleton) loading in sw-GS/PW ensures high paraffin wax content and thus high latent heat retention, which contributes to highly efficient energy storage and release. As shown in Fig. 4e, f, sw-GS/PW-2.25 with a low filler loading of 2.25 vol% (~ 5.3 wt%) exhibited a significantly higher thermal conductivity enhancement factor (especially in the longitudinal direction) and superior latent heat retention (~ 90%) when compared with those of PCCs containing low filler loading (< 10 wt%) published in the open literatures [15, 17, 19, 34, 47–62].

Notably, 3D PCCs (GS/PW and sw-GS/PW) showed anisotropic thermal conductivity owing to the specific 3D heat conduction networks. At 2.25 vol% filler loading, sw-GS/PW possessed 28.2% and 57.8% higher cross-plane and in-plane thermal conductivities, respectively, than GS/PW, indicating a more significant enhancement of thermal conductivity in the transverse direction for sw-GS/PW. It confirmed that sw-GS played a dominant role on the improved in-plane thermal conductivity of PCCs, hence redressing, to some extent, their anisotropy in thermal conductive performances. Thus, the 3D spider web-like network structure is superior to both unidirectional aligned and randomly distributed fillers on thermal conductivity enhancement of polymer composites in general.

### Finite Element Simulation (FE Simulation)

**Fig. 5:**
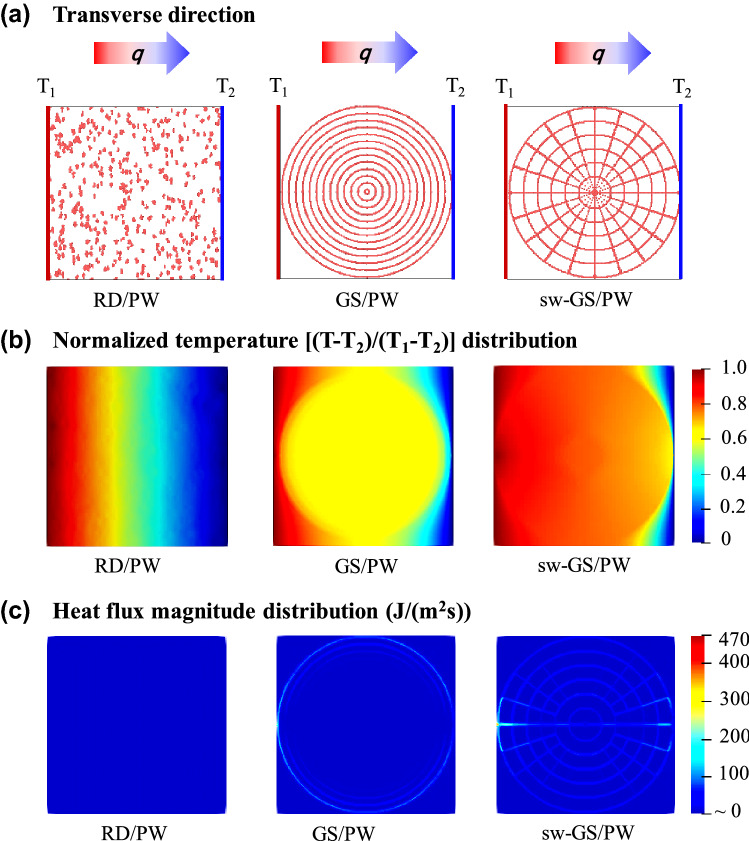
**a** FE simulation for three PCCs. The red (blue) line in the left (right) of the PCCs can be considered a heat source (heat sink). **b** Simulated normalized temperature distribution and **c** heat flux magnitude distribution of nanocomposites based on a heat source and a heat sink. (Color figure online)

To further understand the structural superiority of the sw-GS/PW, especially in the enhancement of transverse thermal conductivity, FE simulations were performed to analyze the thermal transport of the PCCs along the transverse direction during heating. Figure 5 shows the simulated microstructure, temperature and heat flux magnitude distribution of the three PCCs. As shown from the boundary conditions in Fig. 5a, the red (blue) line in the left (right) of the PCCs can be considered as a heat source (heat sink). More FE simulation details can be found in Supplementary Information. In the RD/PW composites (Fig. 5b left), because there is no contact between thermal conductive fillers, poor heat conduction can be seen from the temperature distribution. Also, heat flow is distributed both in the matrix and fillers, which appears weak and evenly distributed. As shown in Fig. 5b, c, heat conduction is better in the GS/PW composites and heat flow is mainly distributed in the concentric annular graphene skeleton. However, scarce connections between concentric annular filler skeleton result in obvious thermal scattering at the interface between filler and polymer matrix, which cause inefficient heat transfer. In the sw-GS/PW composites, the spider web-inspired filler network exhibits interlaced structure that results in many more connections between graphene skeleton, and the temperature on the right surface approaches the temperature of the heat source (Fig. 5b right). In addition, almost all of the heat flow is distributed in the graphene skeleton (Fig. 5c right). Such phenomena demonstrate the spider web-like filler network can significantly reduce the thermal resistance and weaken the phonon scattering between fillers in the transverse direction, which is consistent with the experimental results.

### Thermal Management Application of sw-GS/PW

With the rapid development of new electric vehicles (EVs), the performance, service life and reliability of batteries have attracted much attention since power battery systems are the main source of power of EVs [11, 54, 63]. Currently, lithium-ion batteries are commonly used in this field. However, a large amount of heat would be generated for lithium-ion batteries in the process of continuous running or charging and discharging at high power [11, 54, 63, 64]. Once the heat cannot be dissipated in time, it will produce a significant temperature rise, resulting in deteriorative performance, decreased service life and even serious safety risks of the batteries [17]. Hence, thermal management is of great significance to solving heat safety issues of batteries.

**Fig. 6:**
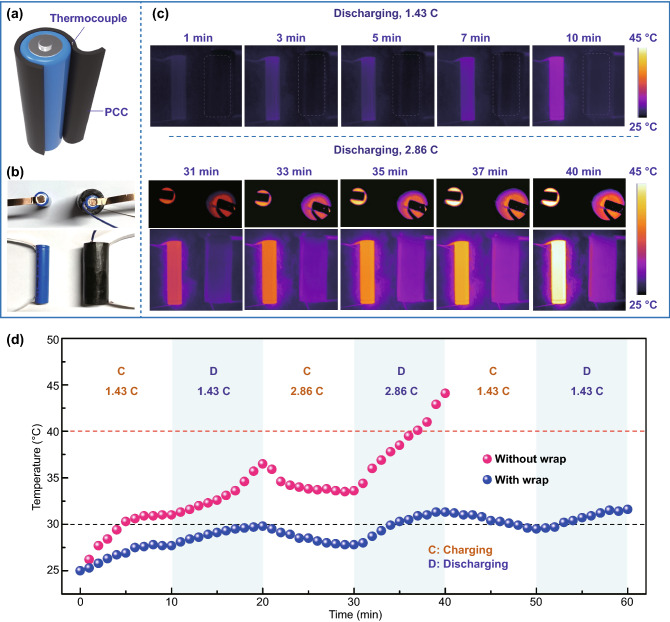
**a** Illustration of integration components for lithium-ion battery with PCC wrap. **b** Optical photographs of lithium-ion battery with/without PCC wrap in a circuit. **c** Infrared thermographs of lithium-ion battery with/without wrap during discharge at the rate of 1.43 C and 2.86 C. **d** Surface temperature variation curves of lithium-ion battery with/without wrap during charging and discharging

In this work, sw-GS/PW-2.25 was used as a thermal management material to regulate the operating temperature of commercial lithium-ion batteries. As shown in Figs. 6a and S7, the PCC was wrapped around the outside of a battery, and a thermocouple device was embedded within. Electrodes were attached to the two ends of the wrapped battery and the integrated device was then connected to a circuit (Fig. 6b). For comparison, an identical battery without the PCC wrap was joined to another electric circuit. Surface temperature changes of the two batteries under the same working conditions were recorded by using a thermal infrared camera. Since the heat generated by a battery during discharge would be much higher than that during charge, only the surface temperature change of the two batteries during discharge was recorded as thermal infrared images in Fig. 6c. It can be seen that at the discharge rate of 1.43 C, the surface temperature of the unwrapped battery was obviously higher than that of the wrapped battery. At an increased discharge rate of 2.86 C, the surface temperature difference between these two batteries became more remarkable. Especially, the surface temperature curves in Fig. 6d showed that after continuous charging and discharging at the rate of 1.43 C and 2.86 C for 40 min, the surface temperature of the PCC wrapped battery was only 31.3 °C, whereas that of the unwrapped battery was 44.1 °C. To assess the potential of sw-GS/PW for regulation of working temperature of the battery, the wrapped battery was allowed to charge/discharge at the rate of 1.43 C continuously and its surface temperature was always maintained lower than 35 °C. Even at a higher charging/discharging rate (2.86 C), results showed that the surface temperature of the wrapped battery remained low and evenly distributed. Also, the paraffin wax used in this work has two phase transition peaks at around 30 and 50 °C in the DSC curves, so it is difficult for the temperature of the wrapped batteries to exceed the warning value even if they continue to operate at a higher charge/ discharge rate. Therefore, thermal management can effectively extend the working life of the batteries, simultaneously keeping a good working state and reducing heat risks.

## Conclusions

In this work, a highly oriented 3D concentric annular hydrogel was synthesized using an alkali-induced method and hydrothermal reaction based on the GO liquid crystals. Inspired by the interlaced structure of spider webs, a 3D spider web-like graphene skeleton (sw-GS) was successfully fabricated from the concentric annular hydrogel by radial freeze-casting. These skeletons were then vacuum impregnated by paraffin wax (PW) to process 3D spider web-like structured (sw-GS/PW) phase change composites. It was shown that sw-GS had little effect on the phase transformation behavior of PW, but it endowed sw-GS/PW with good shape stability and thermal stability owing to the rigid support and the absorption effect of the graphene skeleton. The sw-GS/PW composites displayed high cross-plane and in-plane thermal conductivities of 2.58 and 1.78 W m^−1^ K^−1^, respectively, at a low filler loading of 2.25 vol%. Moreover, sw-GS/PW exhibited outstanding performance on thermal management of Li-ion batteries. They reduced significantly the temperature rise during continuous operation of the battery, confirming their broader applications in electronic and electrical devices.

## Supplementary Information

Below is the link to the electronic supplementary material.

Supplementary file1 (PDF 681 KB)
