# Supplementary material for: Spider Web-Inspired Graphene Skeleton-Based High Thermal Conductivity Phase Change Nanocomposites for Battery Thermal Management
Source: Nanomicro Lett. 2021 Aug 18;13:180. doi: 10.1007/s40820-021-00702-7 (PMC8374024; doi:10.1007/s40820-021-00702-7)
Supplement: Supplementary file 1 — Supplementary file1 (PDF 681 KB) [file 40820_2021_702_MOESM1_ESM.pdf]

Supporting Information for

Spider-Web Inspired Graphene Skeleton-Based High Thermal Conductivity Phase Change Nanocomposites for Battery Thermal Management

Ying Lin[1], Qi Kang[1], Han Wei[2], Hua Bao[2], Pingkai Jiang[1], Yiu-Wing Mai[3], Xingyi Huang[1,] *

[1] Department of Polymer Science and Engineering, Shanghai Key Lab of Electrical Insulation and Thermal Ageing, the State Key Laboratory of Metal Matrix Composites, Shanghai Jiao Tong University, Shanghai, 200240, P. R. China

[2] University of Michigan-Shanghai Jiao Tong University Joint Institute, Shanghai Jiao Tong University, Shanghai 200240, P. R. China

[3] Centre for Advanced Materials Technology (CAMT), School of Aerospace, Mechanical and Mechatronic Engineering J07, The University of Sydney, Sydney, NSW 2006, Australia

*Corresponding author. E-mail: xyhuang@sjtu.edu.cn (Xingyi Huang)

Supplementary Figures

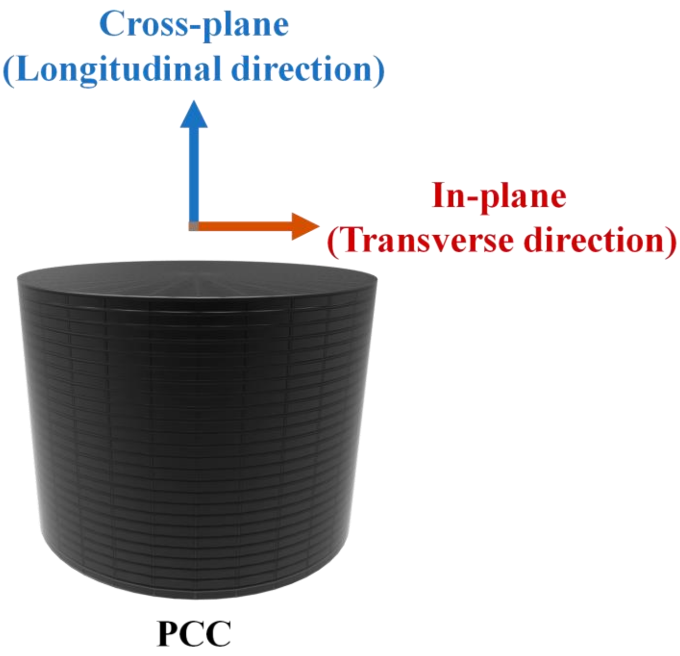

Fig. S1 Cross-plane and in-plane thermal conductivities of PCCs. Spider web-like structure and concentric ring structure are parallel to the in-plane direction.

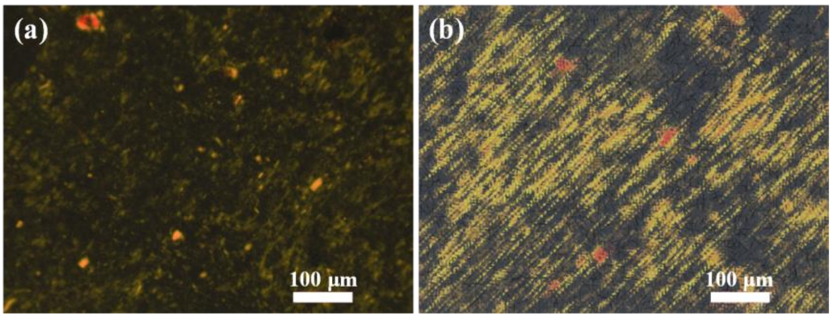

Fig. S2 POM images of 0.5 wt% **a** pure GO suspension and **b** GO suspension with 0.135 M KOH

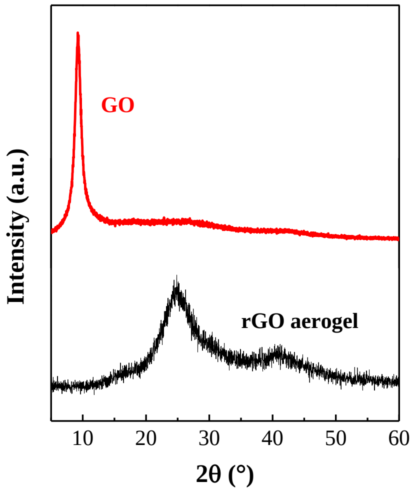

Fig. S3 X-Ray Diffraction (XRD) analysis of GO and freeze-dried rGO hydrogel (aerogel)

As shown in Fig. S3, obvious differences can be observed from XRD results between GO and freeze-dried hydrogel (aerogel). After hydrothermal reaction of GO, the XRD peak of GO at ~10° disappeared, which demonstrated that GO was partially reduced during hydrothermal reaction [S1].

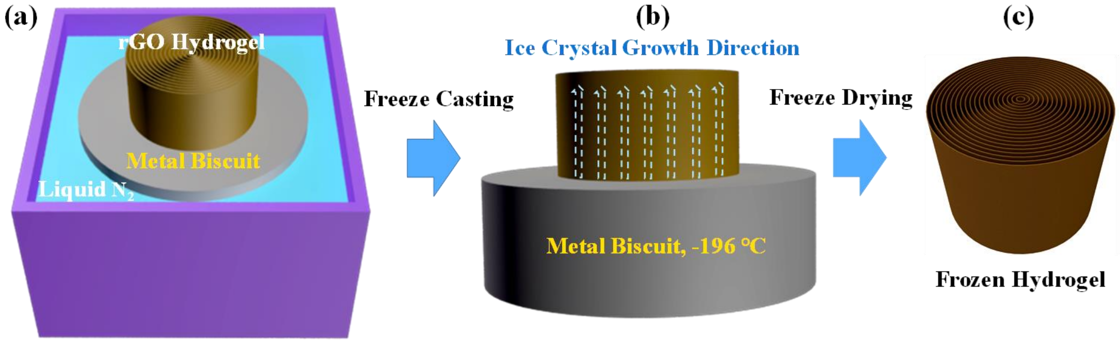

Fig. S4 Schematic diagram of unidirectional freeze casting for GO hydrogel. Schematic diagram of **a** unidirectional freeze casting device, **b** GO hydrogel was placed on a precooled metal block, and **c** frozen GO hydrogel

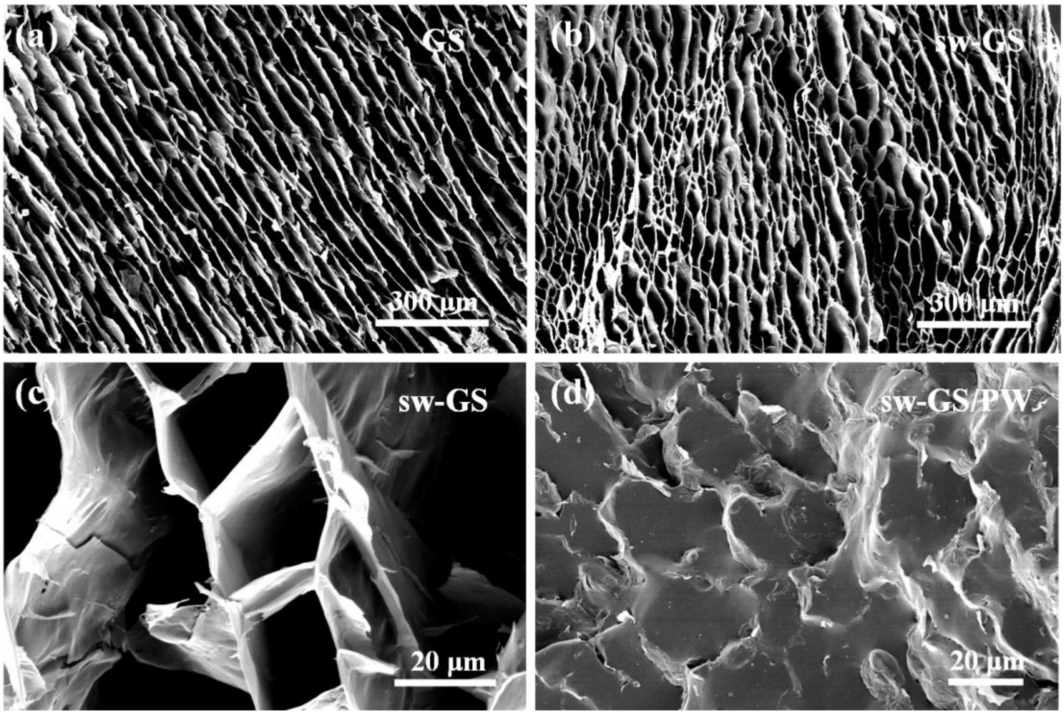

Fig. S5 SEM images of **a** GS, **b**, **c** sw-GS and **d** sw-GS/PW

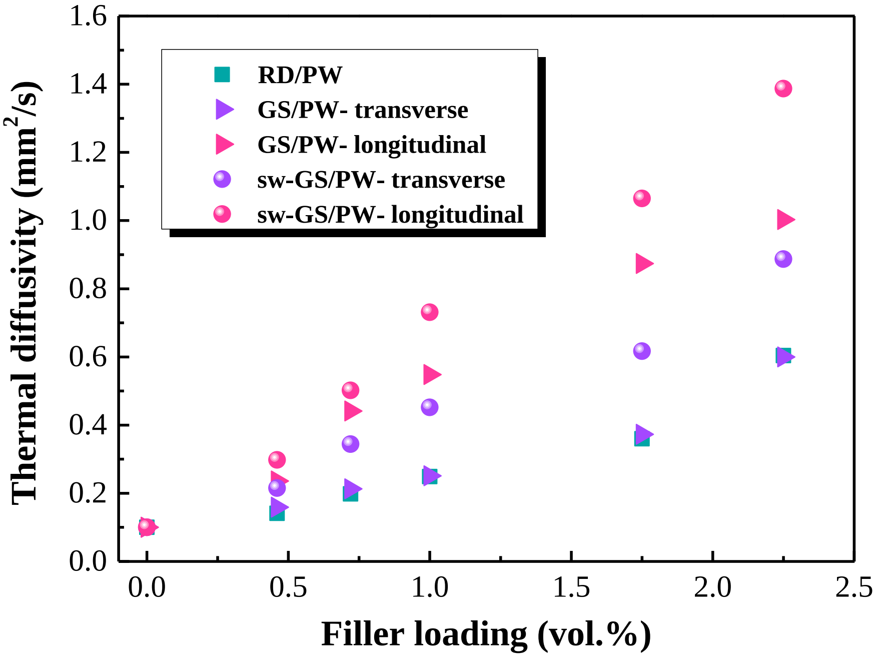

Fig. S6 Thermal diffusivity (at 25 °C) of different PCCs at different filler loading

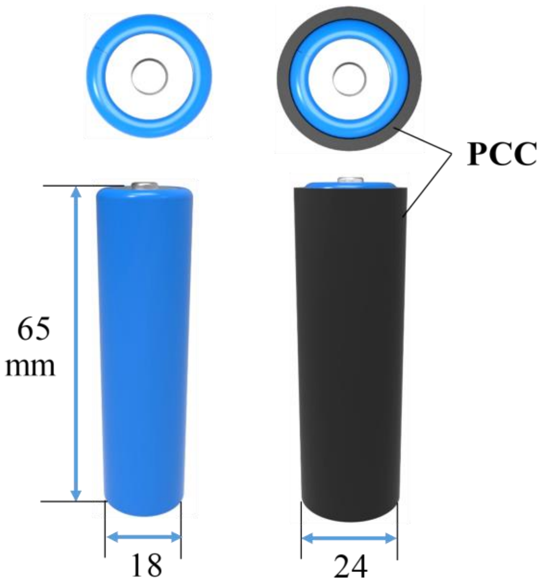

Fig. S7 Schematic diagram of commercial lithium-ion batteries used in thermal management application. Left: battery without wrap; Right: battery with wrap

Finite Element Model

The heat transfer processes of the three PCCs (RD/PW, GS/PW and sw-GS/PW composites) with different filler structures were modeled in Fenics (FEM) [S2]. A simplified model was employed to analyze the temperature and heat flux magnitude distribution of the three PCCs during heating. The heat sources and heat sinks were applied on the left and right surfaces of PCCs, respectively. The periodic boundary conditions were applied on the other surfaces. According to the previous test results and reported results, the κ of graphene oxide sheets and paraffin wax were 350 W/m·K and 0.19 W/m·K, respectively. The filler (skeleton) content of three PCCs was ~10 vol%. In addition, as shown in Fig. S8, the transverse thermal conductivity values of the PCCs based on the model clearly followed the tendency of sw-GS/PW > GS/PW > RD/PW, which is different from experimental results (sw-GS/PW > RD/PW > GS/PW). This could result from the filler (sw-GS powder) retaining some of the interconnected structure of sw-GS in the RD/PW composites. However, in the FEM simulation model, randomly dispersed GO sheets were simulated as the fillers within the RD/PW composites.

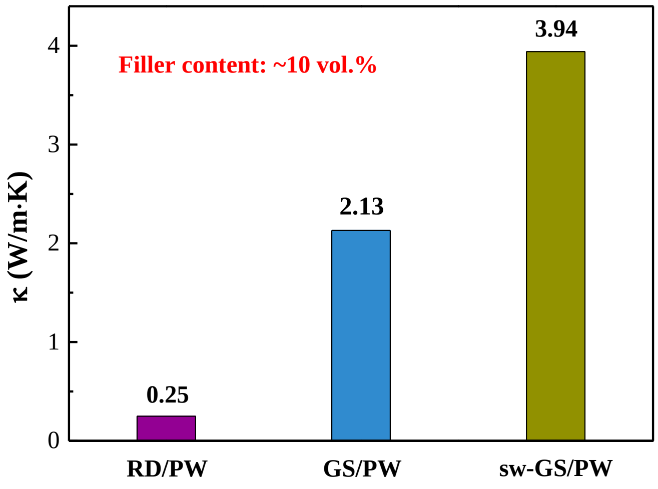

Fig. S8 Simulated thermal conductivities of the PCCs based on the simulation model. The filler (skeleton) content is ~10 vol%.

Supplementary References

[S1] Y. Wang, S. Chou, H. Liu, S. Dou, Reduced graphene oxide with superior cycling stability and rate capability for sodium storage. Carbon **57**, 202-208 (2013). https://doi.org/10.1016/j.carbon.2013.01.064

[S2] M. S. Alnæs, J. Blechta, J. Hake, A. Johansson, B. Kehlet et al., The fenics project version 1.5. Archive of Numerical Software **3**, 9-23 (2015). https://doi.org/10.11588/ans.2015.100.20553
